# Supplementary material for: Whole-brain opto-fMRI map of mouse VTA dopaminergic activation reflects structural projections with small but significant deviations
Source: Transl Psychiatry. 2022 Feb 14;12:60. doi: 10.1038/s41398-022-01812-5 (PMC8844000; doi:10.1038/s41398-022-01812-5)
Supplement: Supplementary file 1 — Supplementary Materials [file 41398_2022_1812_MOESM1_ESM.pdf]

Supplementary Materials

Onset [s]	Duration [s]	Frequency [Hz]	Pulse Width [s]
182.0	20.0	20.0	0.005
332.0	20.0	20.0	0.005
482.0	20.0	20.0	0.005
632.0	20.0	20.0	0.005
782.0	20.0	20.0	0.005
932.0	20.0	20.0	0.005
1082.0	20.0	20.0	0.005
1232.0	20.0	20.0	0.005

Table S1: Block stimulation protocol, coded "CogB".

Onset [s]	Duration [s]	Frequency [Hz]	Pulse Width [s]
180.0	20.0	20.0	0.005
310.0	20.0	20.0	0.005
480.0	20.0	20.0	0.005
630.0	20.0	20.0	0.005
780.0	20.0	20.0	0.005
930.0	20.0	20.0	0.005
1080.0	20.0	20.0	0.005
1230.0	20.0	20.0	0.005

Table S2: Block stimulation protocol, coded "CogBr".

Onset [s]	Duration [s]	Frequency [Hz]	Pulse Width [s]
192.0	30.0	20.0	0.005
342.0	30.0	20.0	0.005
492.0	30.0	20.0	0.005
642.0	30.0	20.0	0.005
792.0	30.0	20.0	0.005
942.0	30.0	20.0	0.005
1092.0	30.0	20.0	0.005
1242.0	30.0	20.0	0.005

Table S3: Block stimulation protocol, coded "CogBl".

Onset [s]	Duration [s]	Frequency [Hz]	Pulse Width [s]
180.0	8.0	20.0	0.005
330.0	10.0	20.0	0.005
480.0	12.0	20.0	0.005
630.0	14.0	20.0	0.005
780.0	16.0	20.0	0.005
930.0	28.0	20.0	0.005
1080.0	20.0	20.0	0.005
1230.0	22.0	20.0	0.005

Table S4: Block stimulation protocol, coded "CogBm".

Onset [s]	Duration [s]	Frequency [Hz]	Pulse Width [s]
150.0000	20.0	15.0	0.005
280.0000	20.0	25.0	0.005
410.0000	20.0	15.0	0.010
540.0000	20.0	25.0	0.010
670.0000	20.0	15.0	0.005
799.9999	20.0	25.0	0.005
930.0000	20.0	15.0	0.010
1060.0000	20.0	25.0	0.010
1190.0000	20.0	15.0	0.005
1320.0000	20.0	25.0	0.005
1450.0000	20.0	15.0	0.010
1580.0000	20.0	25.0	0.010

Table S5: Block stimulation protocol, coded "CogMwf".

Onset [s]	Duration [s]	Frequency [Hz]	Pulse Width [s]
190.0	0.8	25.0	0.005
192.0	0.8	25.0	0.005
194.0	0.8	25.0	0.005
196.0	0.8	25.0	0.005
290.0	0.8	25.0	0.005
292.0	0.8	25.0	0.005
294.0	0.8	25.0	0.005
296.0	0.8	25.0	0.005
390.0	0.8	25.0	0.005
392.0	0.8	25.0	0.005
394.0	0.8	25.0	0.005
396.0	0.8	25.0	0.005
490.0	0.8	25.0	0.005
492.0	0.8	25.0	0.005
494.0	0.8	25.0	0.005
496.0	0.8	25.0	0.005
590.0	0.8	25.0	0.005
592.0	0.8	25.0	0.005
594.0	0.8	25.0	0.005
596.0	0.8	25.0	0.005

Table S6: Phasic stimulation protocol, coded "CogP".

Onset [s]	Duration [s]	Frequency [Hz]	Pulse Width [s]
50.0	1.0	20.0	0.005
90.0	1.0	20.0	0.005
130.0	1.0	20.0	0.005
170.0	1.0	20.0	0.005
210.0	1.0	20.0	0.005
250.0	1.0	20.0	0.005
290.0	1.0	20.0	0.005
330.0	1.0	20.0	0.005
370.0	1.0	20.0	0.005
410.0	1.0	20.0	0.005
450.0	1.0	20.0	0.005
490.0	1.0	20.0	0.005
530.0	1.0	20.0	0.005
570.0	1.0	20.0	0.005
610.0	1.0	20.0	0.005

Table S7: Phasic stimulation protocol, coded "JPogP".

In a linear modelling of the implant coordinate variables, the VTA mean t statistic is found sensitive only to the stimulation protocol category ($F_{1,59} = 57.3$, $p = 2.92\times10^{-10}$), but not the stimulation target depth ($F_{1,59} = 0.48$, $p = 0.49$), the stimulation target posteroanterior (PA) coordinates ($F_{1,59} = 0.59$, $p = 0.45$), and the interaction of the depth and PA target coordinates ($F_{1,59} = 0.48$, $p = 0.49$).

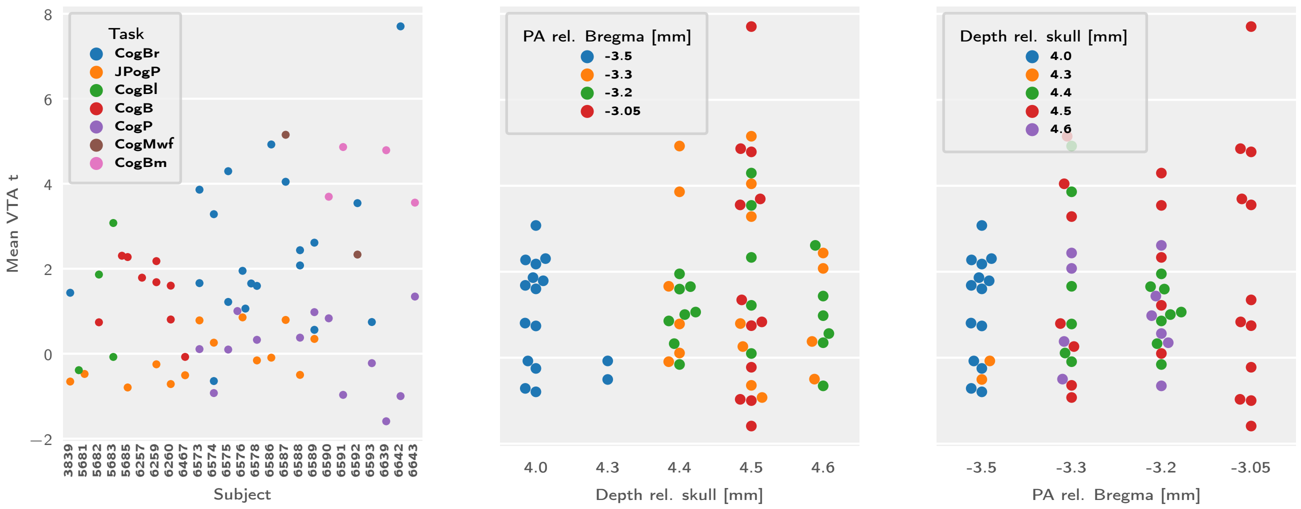

Figure S1: Multifactorial (depth and posteroantior) implant coordinate comparisons of signal intensity in the VTA region of interest. Protocols coded as in tables S1 to S7.

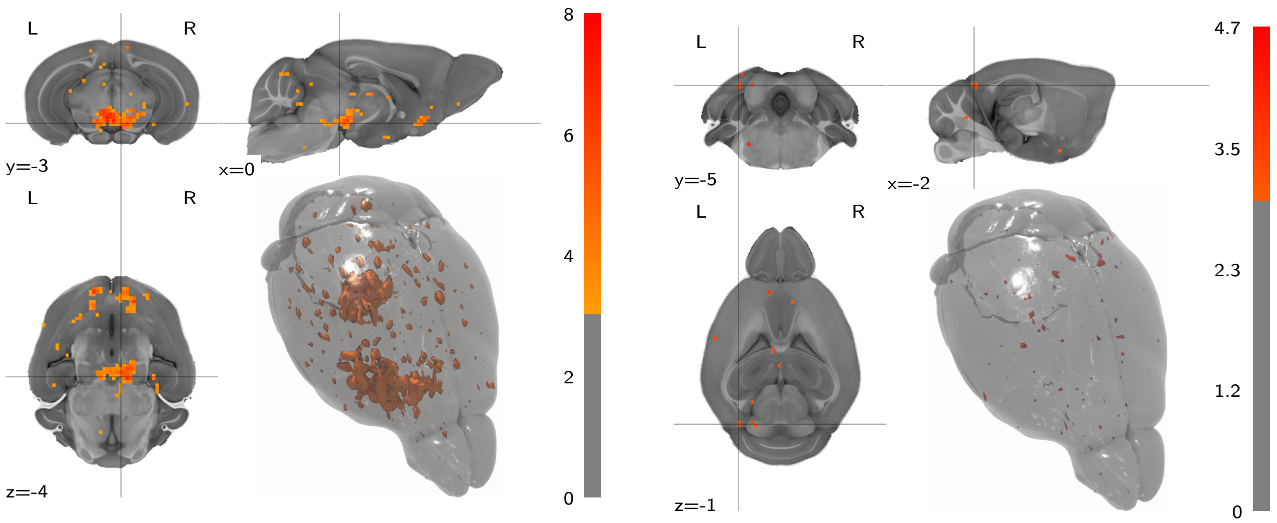

(a) Block stimulation, slices centered on largest cluster.

(b) Phasic stimulation, slices centered on largest cluster.

Figure S2: No negative activation patterns are salient upon block VTA stimulation, and no coherent activation patterns of any sort after phasic VTA stimulation. Depicted are t-statistic maps (thresholded at $|t| \geq 3$) of second-level analyses, divided by stimulation category and binning all implant coordinates. Slices are centered on the VTA coordinates (RAS $= 0.5/-3.2/-4.5$) and the largest cluster, respectively. All maps are adjusted for the wild type control stimulation effects.

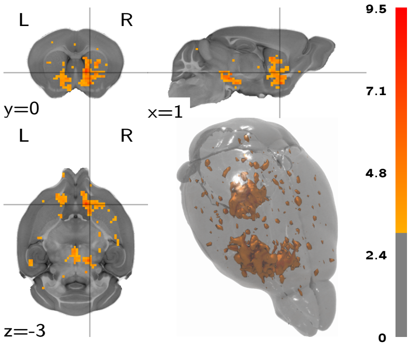

(a) Block stimulation of best implant category group (according to VTA activation), centered on largest cluster.

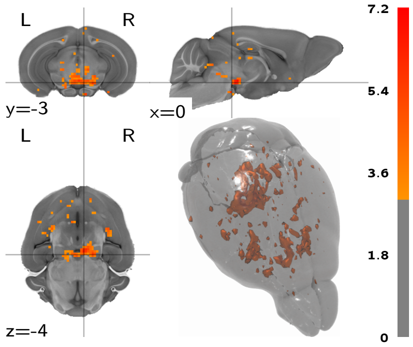

(b) Block stimulation of rejected implant category group (according to VTA activation), centered on VTA.

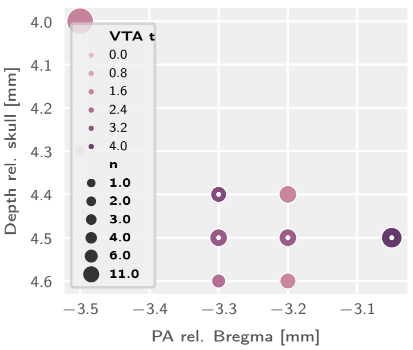

(c) VTA activation-based implant coordinate classification (dotted markers indicate best category).

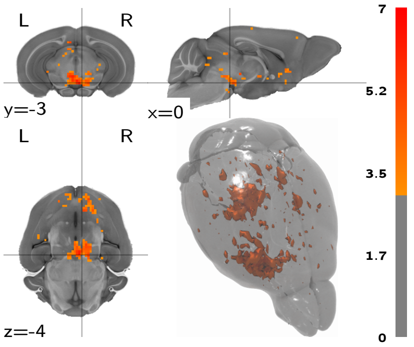

(d) Block stimulation of rostralmost implant category group, centered on VTA.

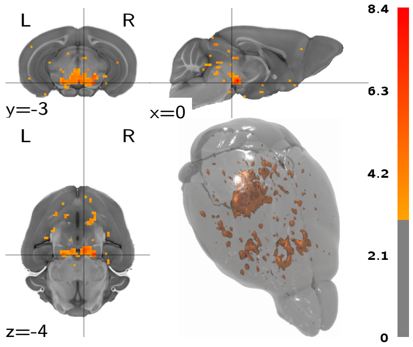

(e) Block stimulation of caudalmost implant category group, centered on largest cluster.

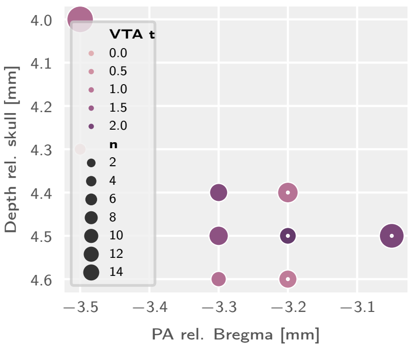

(f) PA-coordinate implant classification (dotted markers indicate rostralmost category).

Figure S3: PA-coordinate-based classification does not show a better projection segmentation than block trial-based classification. Depicted are t-statistic maps (centerd on largest cluster, thresholded at t $\geq$ 3) of the second-level analysis for block stimulation protocols, divided into best and rejected **(a, b)**, or rostralmost and caudalmost **(d, e)**. All maps are adjusted for the wild type control stimulation effects.

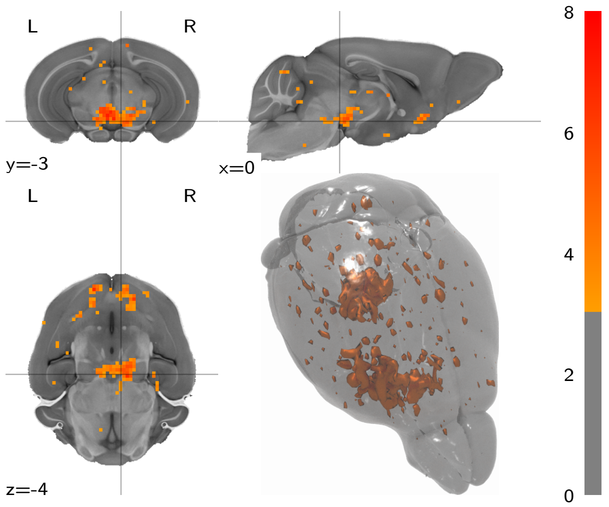

(a) Block stimulation of best implant category group

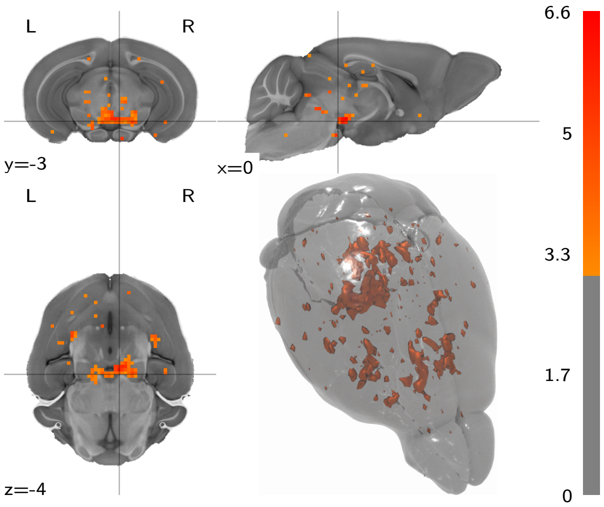

(b) Block stimulation of rejected implant category group.

Figure S4: The uncorrected population-level response to block stimulation does not qualitatively differ from the wild type control corrected results in figs. 3a and 3c. Depicted are wildtype-control-uncorrected t-statistic maps (thresholded at t $\geq$ 3) of the second-level analysis for block stimulation protocols, divided by implant category group. Slices are centered on the VTA region of interest.

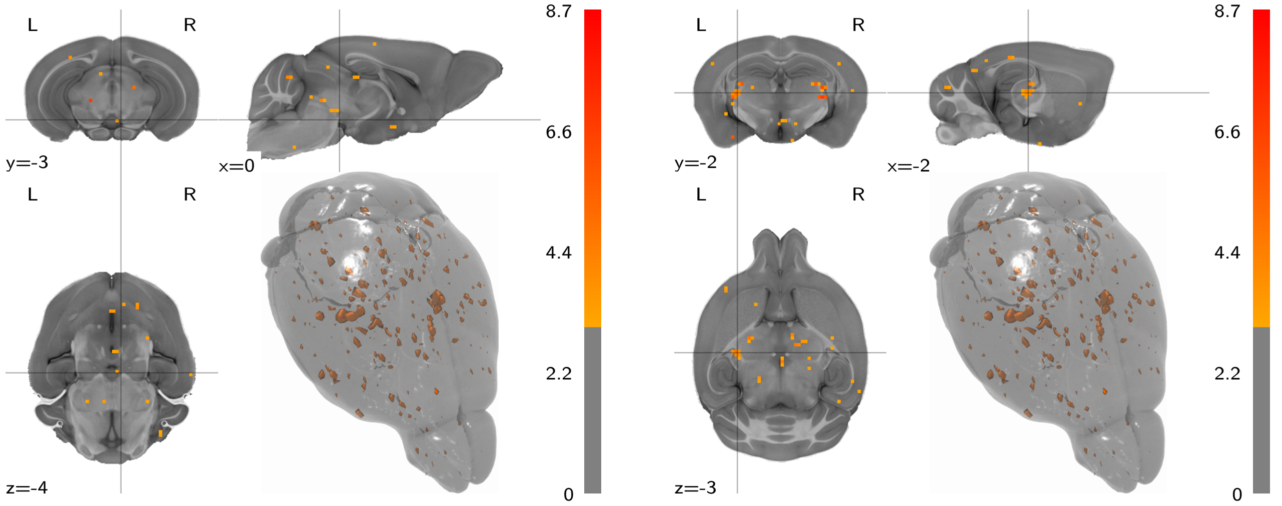

(a) Slices centered on VTA. **(b)** Slices centered on largest cluster.

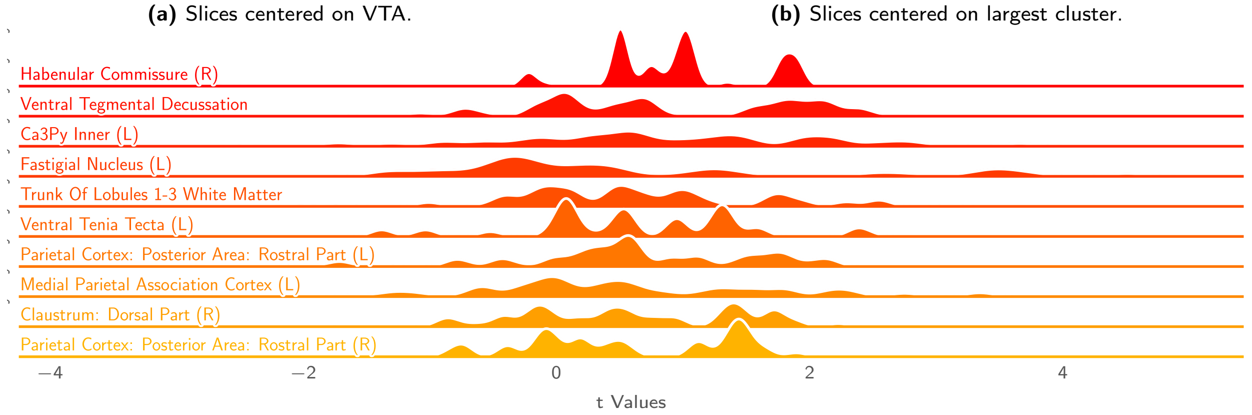

(c) Distribution densities of t-statistic values in the 10 most strongly activated areas.

Figure S5: Block stimulation in wild type control animals produces no large activation clusters, yet scattered activation hints at some visual excitation and heating artefacts. Depicted are volumetric population t-statistic maps **(a, b)** — thresholded at $t \geq 3$, as well as a break-down of activation along atlas parcellation regions **(c)**.

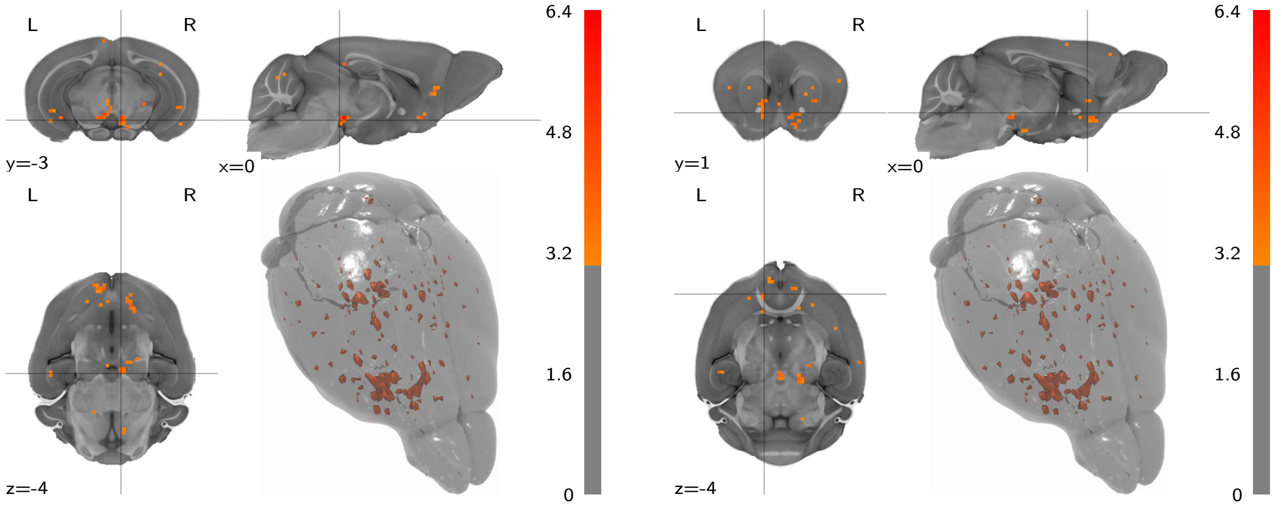

(a) Slices centered on VTA. **(b)** Slices centered on largest cluster.

Figure S6: Depicted are t-statistic maps (thresholded at $t \geq 3$) of the second-level analysis for block stimulation task VTA seed functional connectivity, observed in the best implant category, corrected for the negative control baseline. Slices are centered on the VTA coordinates (RAS $= 0.5/-3.2/-4.5$) and the largest cluster, respectively. This comparison is only provided for the sake of completeness and analogy with the stimulus-evoked analysis. Conceptually this comparison is not of primary interest, since seed-based functional connectivity attempts to include precisely the baseline functioning of the system into the evaluation.

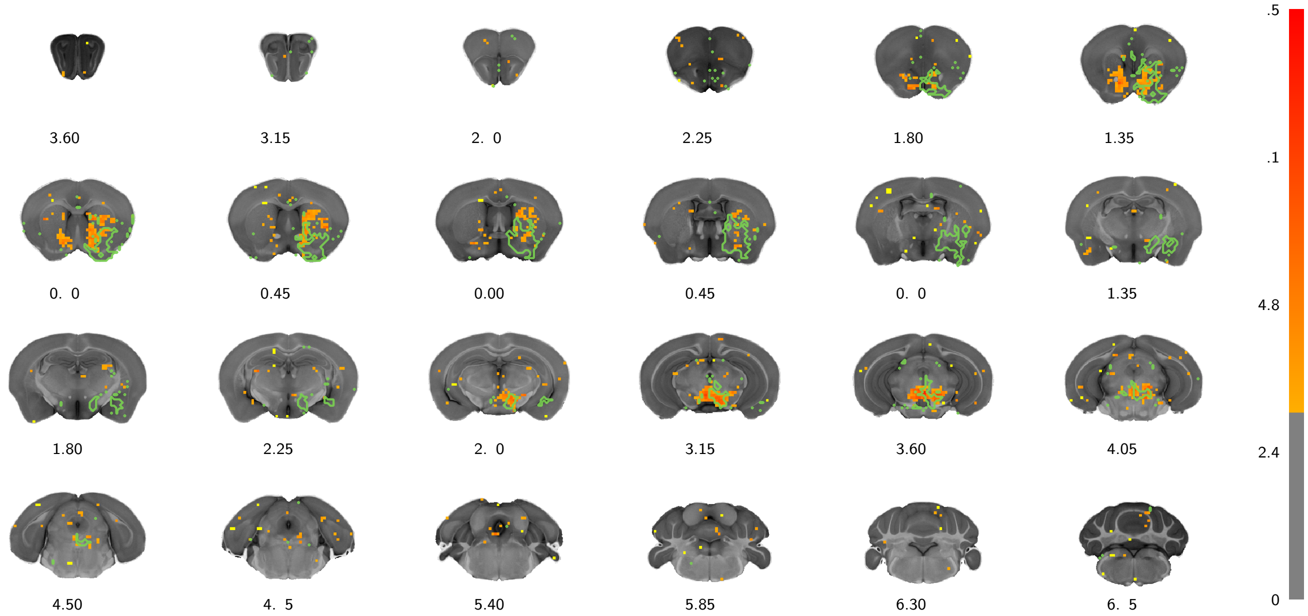

Figure S7: Coronal slice overlay, showing the VTA functional activation t-statistic heatmap (as in **fig. 3a**), and the VTA structural projection outline, both thresholded at t $\geq$ 3. Interpretation of this figure as showcasing a complementarity in the patterns is cautioned, as qualitative inspection of thresholded data does not accurately capture variation in the statistic distributions. For statements regarding the compariosn of functional activation and structural projection, figs. 4a to 4c are more suitable.
